# Persistence of *Borrelia burgdorferi* in Rhesus Macaques following Antibiotic Treatment of Disseminated Infection

**DOI:** 10.1371/journal.pone.0029914

**Published:** 2012-01-11

**Authors:** Monica E. Embers, Stephen W. Barthold, Juan T. Borda, Lisa Bowers, Lara Doyle, Emir Hodzic, Mary B. Jacobs, Nicole R. Hasenkampf, Dale S. Martin, Sukanya Narasimhan, Kathrine M. Phillippi-Falkenstein, Jeanette E. Purcell, Marion S. Ratterree, Mario T. Philipp

**Affiliations:** 1 Divisions of Bacteriology & Parasitology, Tulane National Primate Research Center, Tulane University Health Sciences Center, Covington, Louisiana, United States of America; 2 Comparative Pathology, Tulane National Primate Research Center, Tulane University Health Sciences Center, Covington, Louisiana, United States of America; 3 Veterinary Medicine, Tulane National Primate Research Center, Tulane University Health Sciences Center, Covington, Louisiana, United States of America; 4 Center for Comparative Medicine, Schools of Medicine and Veterinary Medicine, University of California Davis, Davis, California, United States of America; 5 Section of Rheumatology, Department of Internal Medicine, Yale University School of Medicine, New Haven, Connecticut, United States of America; Hopital Raymond Poincare - Universite Versailles St. Quentin, France

## Abstract

The persistence of symptoms in Lyme disease patients following antibiotic therapy, and their causes, continue to be a matter of intense controversy. The studies presented here explore antibiotic efficacy using nonhuman primates. Rhesus macaques were infected with *B. burgdorferi* and a portion received aggressive antibiotic therapy 4–6 months later. Multiple methods were utilized for detection of residual organisms, including the feeding of lab-reared ticks on monkeys (xenodiagnosis), culture, immunofluorescence and PCR. Antibody responses to the *B. burgdorferi*-specific C6 diagnostic peptide were measured longitudinally and declined in all treated animals. *B. burgdorferi* antigen, DNA and RNA were detected in the tissues of treated animals. Finally, small numbers of intact spirochetes were recovered by xenodiagnosis from treated monkeys. These results demonstrate that *B. burgdorferi* can withstand antibiotic treatment, administered post-dissemination, in a primate host. Though *B. burgdorferi* is not known to possess resistance mechanisms and is susceptible to the standard antibiotics (doxycycline, ceftriaxone) *in vitro*, it appears to become tolerant post-dissemination in the primate host. This finding raises important questions about the pathogenicity of antibiotic-tolerant persisters and whether or not they can contribute to symptoms post-treatment.

## Introduction

Lyme borreliosis is caused by the spirochetes of the *Borrelia burgdorferi* sensu lato species complex. The clinical progression of Lyme borreliosis may be divided into early-localized, early-disseminated, and late stages. During the early-localized phase, the disease's most prevalent sign is an erythematous skin rash known as erythema migrans. Subsequently, patients may develop early-disseminated disease with dermatologic, rheumatologic, cardiac, and neurologic involvement. Patients with late disease present chiefly with arthritis or with neurologic manifestations [1]. The Infectious Diseases Society of America (IDSA) has issued guidelines for Lyme borreliosis therapy [2]. Signs and symptoms are usually successfully ameliorated with antimicrobial therapy. However, some patients continue to have persistent subjective complaints [3], [4] while a few patients fail to respond to antibiotic therapy, as made evident by signs of persistent infection [2], [5]. The response to treatment in patients with late manifestations is typically slower [2] and sometimes remains incomplete.

Post-treatment Lyme disease syndrome (PTLDS) is a condition that occurs in some patients after treatment for Lyme borreliosis. The cause of PTLDS is currently unknown but prolonged antibiotic therapy does not seem to be helpful [6], [7]. Objective evaluation of this phenomenon in humans is complicated by the difficulty in obtaining a patient population with confirmed Lyme borreliosis treated post-dissemination, and the vague, non-specific symptoms (fatigue, headache, memory and concentration difficulties, myalgias and arthralgias) with which PTLDS patients present. In addition, reliable procedures to determine that infection has been cleared from Lyme disease patients have not been established.

The C6 ELISA detects antibodies to a region of the *B. burgdorferi* VlsE lipoprotein that is immunogenic in infected individuals and common to all infectious variants tested thus far. Not only is the C6 test among the most reliable in terms of accuracy, but it is also a serologic test for Lyme disease that has been used experimentally as a predictor of treatment outcome [8]. In patients with PTLDS, anti-C6 titers were found to generally persist at a low level compared to acute patient titers [9]. To date, the experimental assessment, in animals, of antibiotic treatment effectiveness, measured by the presence or absence of spirochetes, correlated with C6 serologic test reactivity has not been reported.

Signs and symptoms of putative failure of antibiotic treatment in late disease or ineffectiveness of repeated treatment in patients with PTLDS may be formally attributed to several causes, including: 1) spirochetes that persist in the tissues, likely in small numbers, inaccessible or impervious to antibiotic; 2) inflammatory responses to residual antigens from dead organisms; or 3) autoimmune responses, possibly elicited by antigenic mimicry [10].

In an effort to gain insight into the viability of these hypotheses, we designed two experiments in which we respectively assessed the efficacy of two regimens of ceftriaxone and/or doxycycline treatment in rhesus macaques commencing at 4–7 months of infection with *B. burgdorferi*. Rhesus macaques were chosen because of the ability of this animal model to reproduce many of the key signs of human Lyme disease, including neuroborreliosis [11], [12], [13], [14], [15], [16], [17], [18], [19], [20], [21], [22] and because of the similarity between the available pharmacokinetics data for ceftriaxone and doxycycline in rhesus macaques and in humans [23], [24], [25], [26]. Our results confirm that spirochetes are capable of persisting in treated nonhuman primate hosts. We discuss the possible mechanisms and need for further inquiry into this phenomenon.

## Results

**Figure 1:**
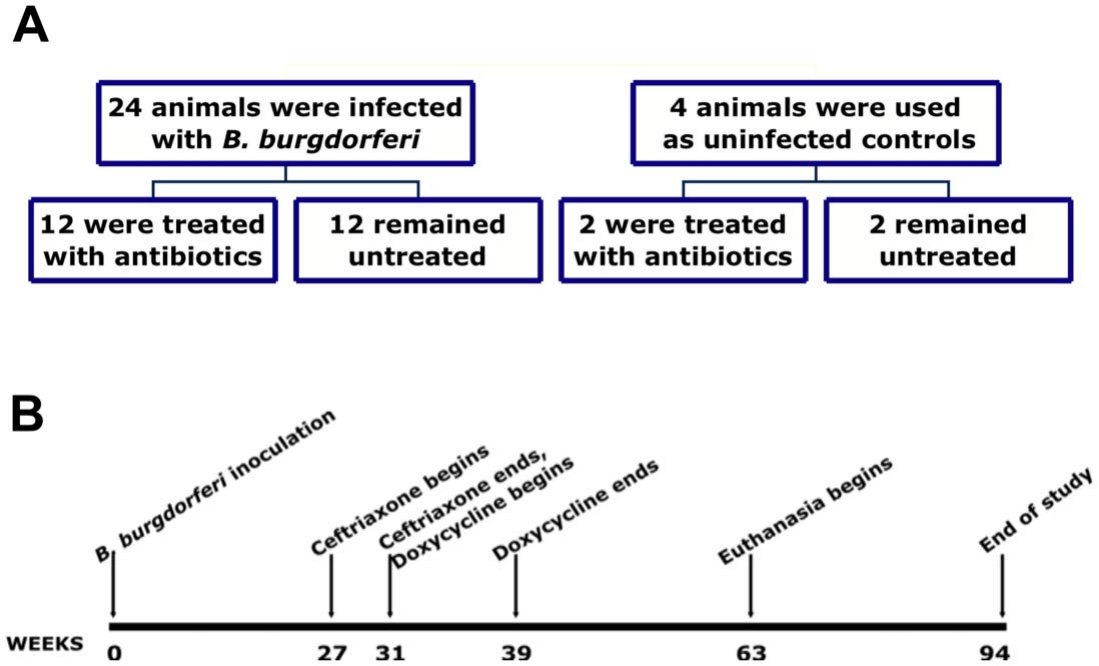
Experimental design for assessment of treatment efficacy in the late, disseminated phase of infection (Experiment 1). A) diagram of animal groups, showing inoculation (*B. burgdorferi* or sham) and treatment groups (treated with antibiotics or untreated); and B) the time line of the study.

We performed two separate experiments to assess post-treatment persistence by *B. burgdorferi in* nonhuman primates with treatment administered at different phases of disseminated infection. Both experiments involved infection, treatment post-dissemination, serology and detection of spirochetes in tissues. The two varied in the number of animals, *B. burgdorferi* strain used, time interval prior to treatment, antibiotic treatment regimen, and detection methods. The first (Experiment 1) was aimed at evaluating animals treated at the late disseminated phase of infection and the treatment regimen was chosen to correspond to the regimen used to treat human PTLDS patients in a clinical evaluation of treatment for this population [6]. The outline for Experiment 1 is depicted in Figure 1.

### Experiment 1: Antibiotic treatment in the late, disseminated phase of infection

#### Evidence of spirochetal infection

During the first 4 weeks post-inoculation (PI), skin biopsies were taken weekly to assess infection by culture. All of the *B. burgdorferi*-inoculated animals yielded culture-positive skin biopsies. In addition, antibody in serum samples that were obtained just prior to the initiation of antibiotic treatment yielded both a positive *B. burgdorferi* whole-cell antigen ELISA, and a positive C6 ELISA in all of the inoculated animals (not shown).

#### Evidence that doxycycline had reached the MIC in serum of treated animals

Serum specimens were obtained from 7 rhesus macaques that were treated with doxycycline as per the regimen of Experiment 1, and 50 µL of each specimen was tested, as described in the Materials and Methods section, for relative concentration. The mean doxycycline concentration at the peak time (2 h) was 2.071±0.431 mg/L or 6.7-fold the MIC of 0.31 mg/L. The median concentration was 2.2 mg/L (range 1.5–2.7). In another experiment, *B. subtilis* was used as the indicator strain. Serum specimens were obtained from 5 additional doxycycline-treated animals both at peak and trough times. The mean doxycycline concentration at the peak was 0.630±0.125 mg/L (median 0.60 mg/L, range 0.45–0.75) and at the trough (12 h) it was 0.160±0.082 mg/L (median 0.15, range 0.10–0.30). The overall median value of the peak doxycycline concentration was 1.35 mg/L and ranged from 0.63–2.07 mg/L). Thus, at the peak, the mean concentration of antibiotic exceeded the MIC in these animals by a factor of 2, but not at the trough, when it was 0.52-fold the MIC. The serum concentration of ceftriaxone was not determined. However, the published concentration of ceftriaxone in rhesus plasma after a single intravenous dose of 20 mg/Kg varies from over 200 mg/L at time zero, to about 50 mg/L at 6 h [25]. The published MIC and MBC for ceftriaxone with *B. burgdorferi* sensu stricto (B31) are 0.013 mg/L and 0.050 mg/L, respectively [27]. Therefore, it is likely that the concentration of ceftriaxone in the rhesus circulation remained above the MIC and the MBC for a significant portion, if not all of the 24-h interval between consecutive doses of this antibiotic.

#### Distinct patterns in the antibody response to C6

**Figure 2:**
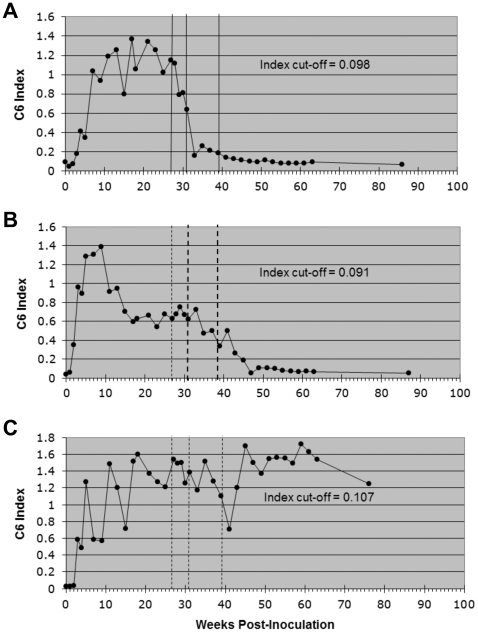
Patterns of antibody response to the C6 peptide in infected animals. The C6 index measured as a function of time PI with *B. burgdorferi* followed three distinct patterns in Experiment 1. Each graph is the longitudinal antibody response to C6 from one representative animal for each pattern. In treated (n = 12) animals (A) the response declined sharply, almost to background levels, within the antibiotic administration periods (solid vertical lines, weeks 27–31 for ceftriaxone, weeks 31–39 for doxycycline). In sham-treated animals (B, C) the response either declined, but independently from the sham treatment intervals, to eventually reach background level (B, n = 7), or oscillated throughout the study period without ever declining steadily (C, n = 5). The pattern in (C) corresponds to the C6-positive animals, indicated in Table 1. The dotted lines in B and C indicate the sham-treatment intervals. The index cut-off value was calculated as described in Material and Methods.

The antibody response to C6 was measured in serially collected serum specimens. In all of the infected animals, the C6 antibody index rose steeply within the first 5–8 weeks post-inoculation (PI) (Figure 2). Thereafter, the responses fit into three patterns, depending on whether the animals were or were not treated with antibiotics. In the treated group, the response declined steadily during the treatment period and reached background levels at the endpoint in all animals (Figure 2A, Table 1). In contrast, the responses of the untreated group remained either largely unchanged (5 out of 12 animals, Figure 2C, scored positive (+) in Table 1), or returned to background levels (7 out of 12 animals, scored negative (−) in Table 1) but not in parallel with the kinetics of the treated group's decline in specific antibody (Figure 2B). The vertical lines in the figures denote the treatment intervals with ceftriaxone and doxycycline (Fig. 2A), or sham (Fig. 2B, C). A comparison of the number of animals with a negative C6 response postmortem in the treated group (11/11) with those in the untreated group (7/12) yielded a statistically significant difference (p = 0.0373).

**Table 1:** Detection of *B. burgdorferi* DNA (PCR), antigen by immunofluorescence assay (IFA), anti-C6 serum antibody (C6), *B. burgdorferi* RNA (RT-PCR), and inflammatory lesions (by Hematoxylin and Eosin stain (H&E)) in all animals at postmortem, 6 to 12 months after the end of the antibiotic treatment (Experiment 1).

ANTIBIOTIC-TREATED					
Animal #	PCR	RT-PCR	IFA	Inflammation(H&E)	C6
AG02	−	−	−	−	−
AG48	−	+ (h, b)	−	−	−
AH77	−	−	+ (h)	−	−
AI97	+ (m, bl, s, l)	+ (h)	+ (m, h)	−	−
AK21	NA	NA	NA	−	−
AL06	−	−	+ (h)	+ (h)	−
AL70	−	−	−	−	−
AL82	−	−	+ (m, h)	+ (h)	−
AM38	−	−	+ (m, h)	+ (h)	−
BF20	−	−	+ (m, h)	−	−
BF21	−	−	−	−	−
BH50	−	+ (h)	+ (h)	−	−
BR97 (uninf.)	−	−	−	−	−
BR99 (uninf.)	−	−	−	−	−

SHAM-TREATED					
Animal #	PCR	RT-PCR	IFA	Inflammation(H&E)	C6
BD71	−	−	+ (h)	−	+
BG03	−	−	+ (h)	−	+
BG48	−	−	+ (m, h)	−	+
BG96	−	−	+ (m, h)	−	−
BH18	−	−	+ (m, h)	−	−
BH19	−	−	−	−	−
BH31	−	+ (h)	+ (m, h)	−	+
BH79	−	−	−	−	−
BI80	−	−	+ (m, h)	−	−
BT02	+ (drg)	−	−	−	−
V517	−	+ (b)	+ (m, h)	−	−
BE40	+ (h)	−	+ (m, h)	−	+
AH79(uninf.)	−	−	−	−	−
V789(uninf.)	−	−	−	−	−

b, brain; bl, bladder; drg, dorsal root ganglia; h, heart; l, lung;

m, meninges; s, spleen.

(uninf.): animals not inoculated with *B. burgdorferi*.

NA: sample not available.

#### Monitoring of infection status postmortem

Infection status postmortem was evaluated by culture, PCR, RT-PCR, gross pathology, histology, and immunofluorescence. All of the animals were assessed, except one of the animals in the treated group (AK21), which died due to anesthetic shock 3 months PI. With regard to culture of spirochetes, one of the treated animals yielded a positive culture and only one of the untreated animals was culture-positive (lung tissue).

With regard to PCR, tissues were assessed for spirochetal DNA by PCR that amplified both the *flaB* and *ospA* genes of *B. burgdorferi*. Among the untreated group, 2 animals were positive (Table 1). Here, spirochetal DNA was found in the dorsal root ganglia for one animal, and in the heart for the other. One of the animals of the treated group was PCR positive (Table 1), and in several organs, including the meninges, bladder, spleen, and lungs. The difference between treated and untreated groups was not statistically significant (p = 1.000).

For detection of *B. burgdorferi* transcript, RNA was extracted from heart and brain specimens. Two of the animals that were not treated with antibiotics were positive, one in heart and the other in brain. Two of the treated animals had detectable spirochetal RNA in the heart, and one additional treated animal was positive for *B. burgdorferi* RNA in both heart and brain (Table 1). The difference was not statistically significant (p = 0.6464).

**Figure 3:**
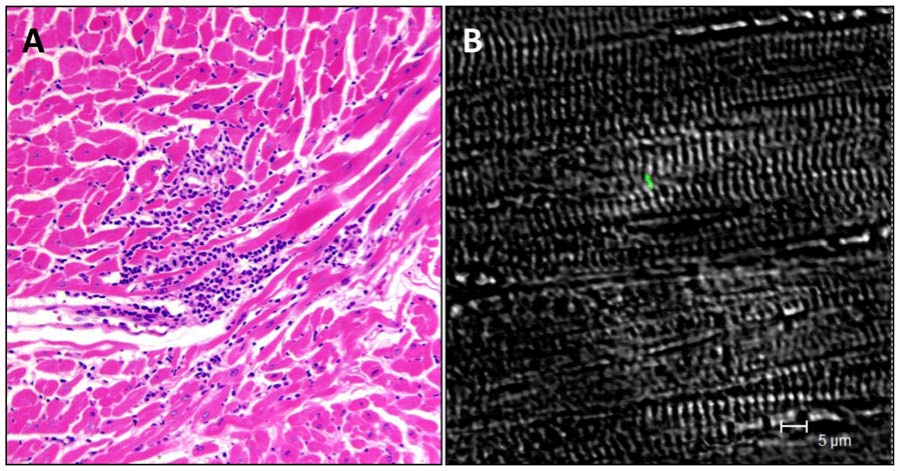
Images of tissue inflammation and *B. burgdorferi* antigen in tissues from animals treated in the late, disseminated phase (Experiment 1). For antigen detection, samples of tissue were stained for fluorescent detection (IFA) with anti-*B. burgdorferi* monoclonal (see Materials and Methods) antibody. (A) Hematoxylin &Eosin stain showing monocytic and lymphocytic infiltrate in a heart section (20×) of a treated animal (AM38). (B) Image of positive IFA staining from the heart tissue of the same animal.

In the assessment of gross pathology, histology, and immunofluorescence, no gross lesions were observed in any of the animals. Fragments of heart and meninges were collected postmortem, and fixed, sectioned and stained for histology and immunofluorescence. Three animals, all of them treated, had moderate to severe inflammatory lesions in the heart (Table 1, Figure 3A). Positive immunofluorescent staining for *B. burgdorferi* appeared as fragments. Serial sections of tissue were cut at 6 µm width, so only bacteria lying perfectly parallel to the section would appear as 12–17 µm in length (the reported length of B31 spirochetes [28] (Figure 3B). We did not find samples of this type. However, there were numerous immunofluorescence-positive specimens both in the untreated and the treated groups (Table 1). The difference was not statistically significant (p = 0.6668). The four animals that were not inoculated with *B. burgdorferi* were negative by this test (Table 1).

### Experiment 2: Antibiotic treatment in the early, disseminated phase of infection

**Figure 4:**
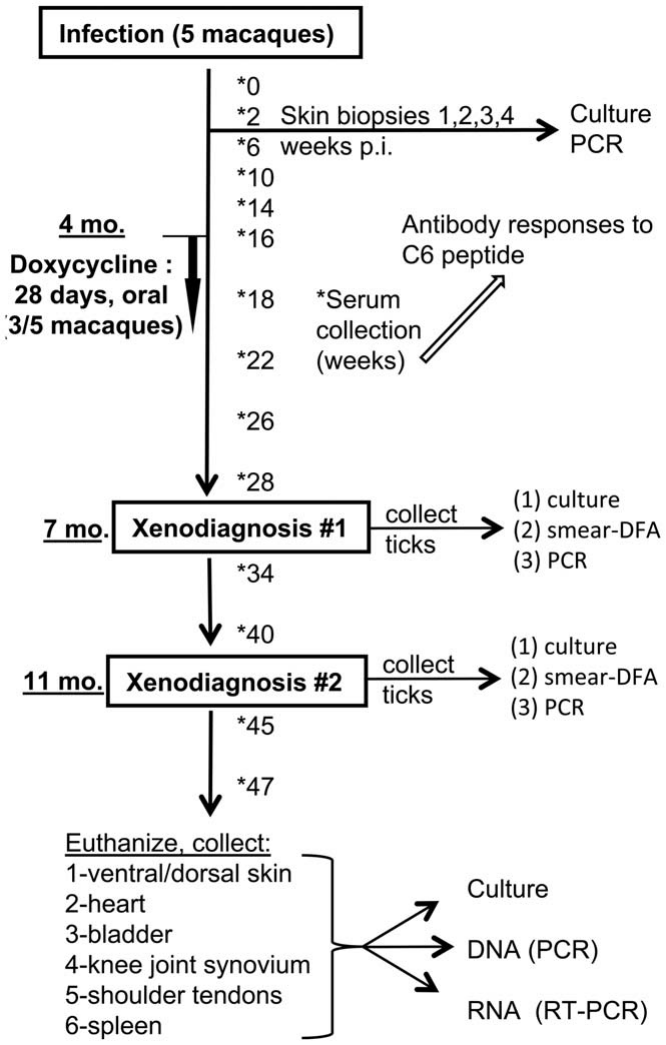
Experimental outline and sample collection for assessment of treatment efficacy in the disseminated phase of infection (Experiment 2).

The second experiment (Experiment 2) focused on animals treated at the early disseminated phase and the duration of the treatment regimen was chosen by the IDSA guidelines [29]. The dose of doxycycline given was markedly higher than in Experiment 1. In addition to molecular methods, xenodiagnosis was used for detection of infection. The experimental outline is depicted in Figure 4.

#### Confirmation of *B. burgdorferi* infection

In Experiment 2, skin biopsies and blood were taken at 1–4 weeks PI. Skin samples were cultured in BSK-H medium and subjected to DNA extraction for *Borrelia*-specific PCR. Seroconversion was tested by ELISA for antibody reactivity to the C6 peptide [30], the Ct peptide (a 51-mer synthetic peptide that reproduces the entire sequence of the C-terminal invariable domain of VlsE from *B. burgdorferi* strain B31) [31] and outer surface protein C (OspC) [32]. Infection was indicated by *Borrelia* DNA detection in skin biopsies and by serology (Table S1). Cultures were kept 12–15 weeks, but none were positive for spirochete growth.

#### Decrease in anti-C6 antibody titers with treatment

We measured the responses to C6 longitudinally to include before, during, and after treatment. The endpoint dilution titers were also determined for the final time point (week 47 for Experiment 2). The C6-specific antibody levels declined in all animals treated with antibiotics (Figure S1). In untreated animals, the anti-C6 responses either leveled off or slowly declined. To determine if antibody titers declined to baseline, the endpoint dilution titers for each of the five animals in Experiment 2 were determined for sera collected before, during, and after treatment. For all of the untreated animals, the titers remained high and the titers from the treated animals declined markedly, and remained at low level even at 27 weeks post-treatment (Table 2).

**Table 2:** Reciprocal end-point dilution titers of anti-C6 antibodies in antibiotic-treated* (**bold**) and untreated macaques infected with *B. burgdorferi* (Experiment 2).

	16 weeks PI	32 weeks PI	47 weeks PI
**GA59**	>2560	640	320
**FK38**	>2560	40	160
**GB56**	>2560	160	320
GC84	>2560	>2560	>2560
FT47	>2560	>2560	>2560

*treatment was given during weeks 16–20.

#### Detection of spirochetes in treated animals by xenodiagnosis

**Figure 5:**
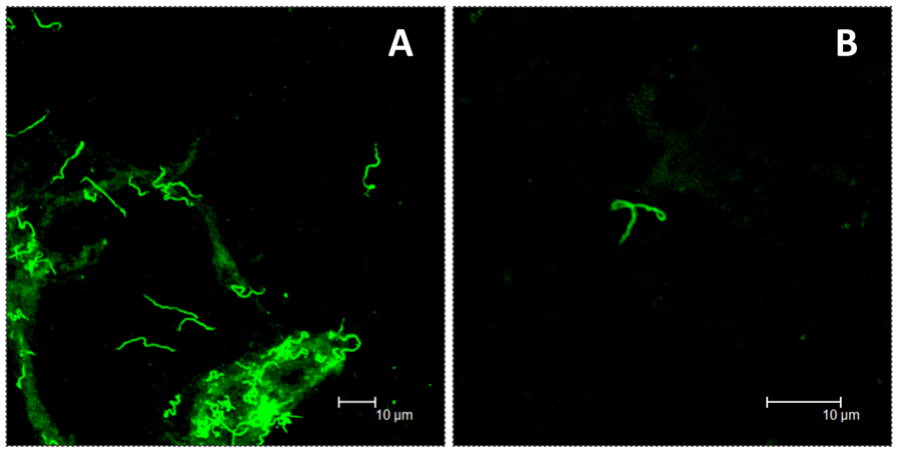
Spirochetes recovered by xenodiagnosis from animals treated in the disseminated phase of infection. Images from direct fluorescent staining of *B. burgdorferi* spirochetes found in xenodiagnostic tick midgut culture (A) or tick midgut preparation (B) from treated animals GA59 and GB56, respectively.

At 7 and 11 months PI, all monkeys were fed upon by *Ixodes scapularis* nymphs for the uptake of persisting spirochetes by xenodiagnosis. The number of nymphs that fed to repletion varied considerably between animals. For the first round, a total of 7, 8, and 11 ticks, respectively, fed on the treated animals, whereas only 5 ticks fed on each of the untreated animals (Table S2). Tick midguts were split into 3 parts for culture, direct fluorescence and for DNA extraction. The probability of recovering spirochetes would be higher from animals upon which more ticks feed. As such, intact spirochetes were detected from the cultured midgut contents (Figure 5A) or directly from tick midgut smears (Figure 5B) of two animals at 7 months PI, both of which had been treated. These animals (GB56 and GA59) also had the most xenodiagnostic ticks feed (Table S2).

#### Detection of spirochetal nucleic acids in tissues

**Figure 6:**
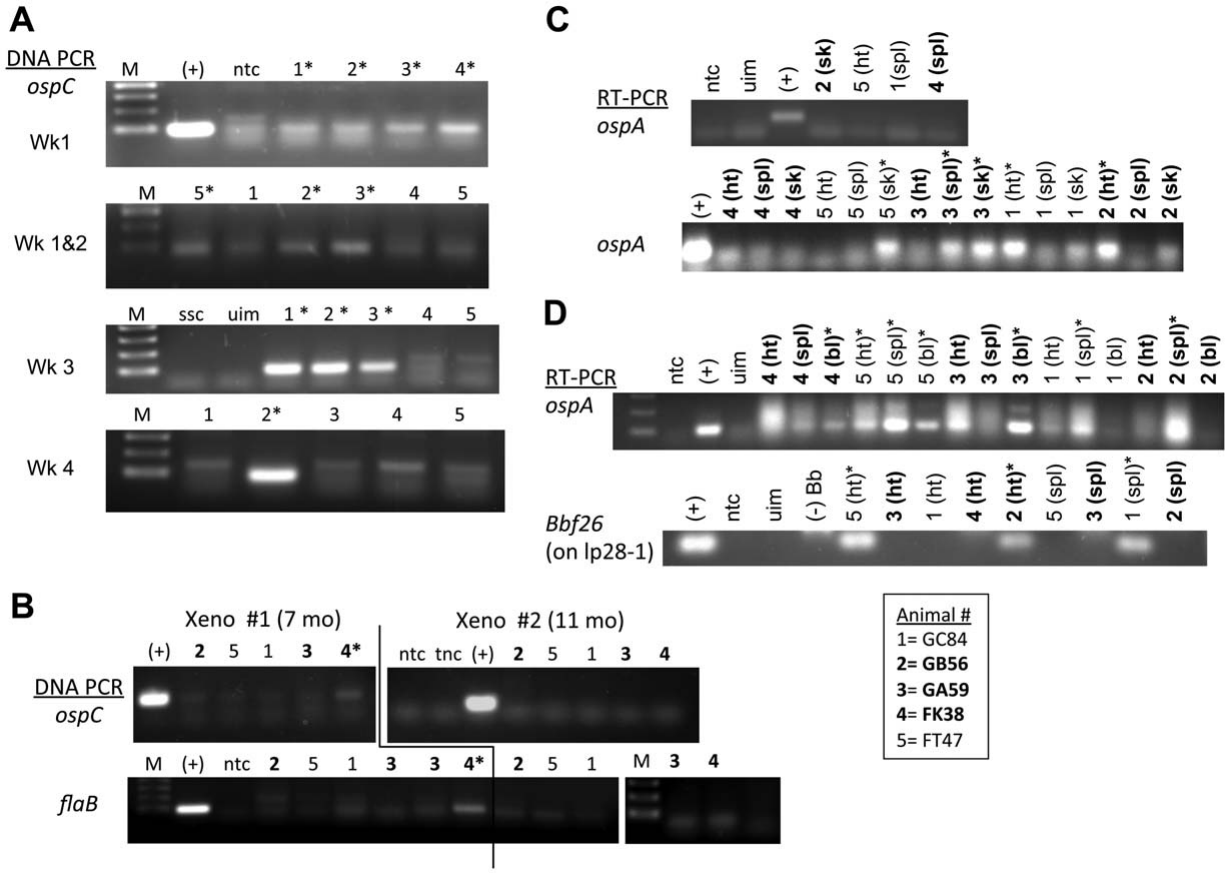
Nucleic acid detection of *B. burgdorferi* (Experiment 2). Detection by PCR or RT-PCR using primers for the indicated genes from: A) skin biopsy samples; B) xenodiagnostic ticks; C) organ tissue culture pellets; and D) directly from tissues known to harbor the spirochetes. Animal numbers in **bold** are of those that were treated. Asterisks indicate clear positive amplimers.sk = skin; h t = heart; bl = bladder; spl = pleen. Labels include: M = marker (100 bp ladder); (+) = *B.burgdorferi*, strain B31 DNA/RNA; ssc = spirochete strain negative control (*Bb* strain JD1 DNA); ntc = no template control; tnc = tick negative control; uim = uninfected monkey DNA/RNA.

For Experiment 2, several *B. burgdorferi* genes were used both for detection of spirochetes (by standard PCR) and for detection of metabolically active spirochetes (by RT-PCR). The detection of genes throughout the course of infection, in tissues, by standard PCR/RT-PCR is shown in Figure 6.

The *ospC* gene was amplified from skin biopsy tissue from each animal in at least one of the four samplings (Figure 6A) shortly after inoculation. These results confirmed infection and specific detection by the *ospC* PCR method. The detection of *B. burgdorferi* nucleic acids by PCR was also performed on xenodiagnostic tick midgut samples (Figure 6B). For treated animal FK38, spirochetal DNA was detected by amplification of *ospC* from midgut contents when organisms were not detected by culture or DFA (Figure 6B).

A few spirochetes grew in cultures of organ tissues collected post-mortem from each animal after >9 weeks, but we were unable to subculture any spirochetes from either treated or untreated animals due to their slow growth. We therefore pelleted these cultures to confirm their identity and test their viability by DNA/RNA analysis. Transcription was detected in culture pellets and the tissues of treated animals, indicating that the bacteria were metabolically active (Figure 6C, D). Figure 6D shows *ospA* transcription detected directly in tissues harvested from treated and untreated animals. We also hypothesized that persistent spirochetes may lose linear plasmid 28-1 (lp28-1), which encodes the VlsE antigen bound by the anti-C6 antibody. Transcription of a lp28-1 gene (*bbf26*) was verified in organ tissue from both untreated animals and one treated animal (Figure 6D).

## Discussion

In some cases, patients who have been treated for Lyme disease experience persistent symptoms. The assertion that further antibiotic treatment is warranted in these cases is a matter of contention and considerable debate [33], [34], [35], [36]. Our results indicate that disseminated spirochetes of two different *B. burgdorferi* strains can persist in the primate host following high dose, or long-lasting antibiotic therapy. In terms of disease, only objective signs of disease post-therapy may be measurable in an animal model. While we did not find gross signs of disease postmortem, in Experiment 1 we did identify heart sections with inflammatory infiltrates in three of the treated animals. In addition, several animals, both treated and untreated showed sections of heart and meninges that were positive by immunofluorescence for *B. burgdorferi*. At the molecular level, *B. burgdorferi* DNA would indicate the presence of organisms, live or dead. The detection of RNA, however, should indicate that those present are metabolically active and thus alive. In Experiment 1, spirochetal DNA and RNA were detected in the tissues of a few animals, independent of treatment. This may reflect a low spirochetal burden, lack of *flaB* transcription [37], and/or seclusion in untested tissues.

In Experiment 2, we were able to recover spirochetes by xenodiagnosis in two of the three treated animals. The inability to recover spirochetes from all five macaques could be in part attributed to the number of ticks that fed, which was markedly smaller for untreated animals (Supplementary Table S2). However, a few slow-growing organisms were recovered by culture from each animal. We detected the *ospA* transcript in culture pellets from tissues of four animals and directly from at least one tissue from each animal. We chose *ospA* because this gene has been shown to be transcribed by host-adapted *B. burgdorferi* [38], in disseminated infection [39], and because of the induction of an anti-OspA response in patients [40] post-dissemination.

The nature of the persistent organisms and the acquisition of tolerance to antibiotics are questions that need to be addressed. The *B. burgdorferi* spirochete is known to invade collagenous tissue as a possible mechanism of immune evasion. It has been postulated that the joint tissues provide a protective niche during antibiotic treatment [41]. Our studies and others [37], [42], however, have not demonstrated a specific predilection for spirochete presence in joints of treated animals. Antibiotic tolerance has been demonstrated *in vitro* with several bacterial species, both gram negative (*E. coli*) and gram positive (*Staphylococcus* spp.). The fact that organisms can persist in the presence of antibiotics such as penicillin and cephalosporins (ceftriaxone) that interfere with cell wall synthesis appears to stem from their ability to enter a dormant, non-dividing state [43], [44], thus avoiding the need for cell wall synthesis to continue growth. The other antibiotic that was used in these experiments is doxycycline. The tetracycline class of antibiotics corrupts translation at the ribosome; therefore, minimal gene expression from dormant organisms may be unaffected. A “persister” phenotype may possibly be responsible for the recalcitrance of persisting spirochetes made evident by previous studies in mice and dogs [37], [42], [45], and by those presented in this report. Perhaps incomplete clearance of bacteria following antibiotic treatment is not a phenomenon unique to *B. burgdorferi*, but one that occurs with other bacterial infections as well. In this case, xenodiagnosis enables detection of otherwise inconspicuous live organisms through acquisition by the natural vector.

To date, the C6 ELISA is the only test in which a decline in antibody titer is statistically associated with outcome of antibiotic treatment [8], [46], [47]. In accord with this finding, we observed a distinct and rapid decline in C6 titer in all antibiotic-treated animals. Not all animals, however, were spirochete-free, so the question of what facet of infection is indicated by anti-C6 antibody titers was brought to the fore. Also, the C6 titers declined in some untreated animals over a long period of time, but not in others, though presence of spirochetes was indicated in C6-negative untreated animals by IFA or PCR/RT-PCR. This is likely due in part to genetic differences in outbred animals. Possible explanations for the lack of correlation between C6 response and presence of spirochetes include: (1) the anti-C6 titer is an indicator of treatment efficacy and the infection is cleared despite the presence of spirochetal genetic material/antigen; (2) organisms persist and the anti-C6 titer does not reflect their presence, perhaps due to loss of plasmid lp28-1 (which encodes VlsE, the parent molecule of C6); or (3) anti-C6 titer declines with a significant reduction in spirochetal burden, but a low number of organisms reside in the host; if these organisms are dormant, then transcription of *vlsE* also may be negligible, minimizing re-stimulation with antigen. The detection of intact organisms ruled out the first explanation and detection of transcript (*bbf26*) from lp28-1 disproves that explanation #2 may be operating exclusively. We therefore favor explanation #3 and seek to determine the level of transcriptional/metabolic activity, and pathogenicity of persistent organisms. If, for example, spirochetes that are recovered by xenodiagnosis from treated animals turned out to be non-pathogenic, this would validate the decline in C6 titer as a measure of successful treatment outcome.

In infected mice treated with tigecycline, transcription of the chromosomally-encoded *B. burgdorferi oppA2* gene was detected in 8 of 9 treated mice, indicating spirochetal viability [37]. Importantly, these studies also indicated that DNA copies of another gene, *ospA*, were present in treated mice, but were significantly fewer than in untreated mice. This result likely reflects a much lower spirochetal burden with treatment, supporting the notion that anti-C6 titer may decline as a function of reduced numbers. The C6 titers in these mice were not determined. Due to the relatively small quantities of bacteria over a large amount of tissue, we were unable to reliably quantify DNA or transcript levels in nonhuman primates. Similarly, the recovery of few spirochetes by tissue culture is aptly a reflection of the rhesus model and not necessarily the treatment [48].

The most pressing question in terms of human disease is whether or not spirochetes remain pathogenic after antimicrobial therapy. Similarly, do spirochetes persist long-term, or are they eventually cleared by the host? Clearly, the phenotype of persistent organisms needs to be elucidated. These studies support the use of the C6 test for diagnosis and measurement post-treatment; however, the absolute quantification of antibody levels may be essential in determining treatment efficacy for PTLDS patients, as low levels (yet above baseline) may indicate presence of residual spirochetes or antigen. Finally, the use of variable and pulse-dosing regimens of antibiotics may improve efficacy [43] and this warrants testing in an appropriate model.

Finally, in these studies we used an artificial mode of inoculation and spirochetal dose. The experimental results must be confirmed with tick-mediated infection, which is our intent. Our studies do however offer proof of the principle that intact spirochetes can persist in an incidental host comparable to humans, following antibiotic therapy. Additionally, our experiments uncover residual antigen associated with inflammatory foci. Whether persistent spirochetes or spirochetal antigen can cause PTLDS remains unanswered.

## Materials and Methods

### Ethics statement

Practices in the housing and care of animals conformed to the regulations and standards of the PHS Policy on Humane Care and Use of Laboratory Animals, and the Guide for the Care and Use of Laboratory Animals. The Tulane National Primate Research Center (Animal Welfare Assurance # A4499-01) is fully accredited by the Association for the Assessment and Accreditation of Laboratory Animal Care-International. The Institutional Animal Care and Use Committee (IACUC) of the Tulane National Primate Research Center approved all animal-related protocols, including the infection, treatment, and tick-feeding procedures used with nonhuman primates. All animal procedures were overseen by veterinarians and their staff. For blood collection, skin biopsy collection and the tick capsule procedures, monkeys were anesthetized with ketamine (10 mg/kg) by intramuscular injection. Animals were humanely euthanized by the veterinary staff at the TNPRC in accordance with endpoint policies. Euthanasia was conducted by anesthesia with ketamine hydrochloride (10 mg/kg) followed by an overdose with sodium pentobarbital. This method is consistent with the recommendation of the American Veterinary Medical Association guidelines.

### Experimental design

In the first experiment (Experiment 1), the treatment regimen was initiated at 27 weeks PI. It included the administration of ceftriaxone for 30 days, followed by 60 days of doxycycline. The latter antibiotic is used primarily to treat early localized and disseminated disease, whereas ceftriaxone is used to treat late disease [2]. Animals were kept for a minimum of 6 months after treatment termination. Tissue samples were collected for 1) *in vitro* culture of *B. burgdorferi*; 2) quantitative real-time PCR and reverse transcriptase (RT)-PCR, for detection of *B. burgdorferi* nucleic acids; and 3) histopathology/immunofluorescence to localize inflammatory lesions and spirochetes in tissues, respectively. The C6 test was also performed on serum specimens collected serially from all of the animals.

In the second experiment (Experiment 2), the treatment regimen began 4 months after inoculation with *B. burgdorferi* and consisted of a 28-day regimen of oral doxycycline, as per the IDSA guidelines for disseminated infection [2]. At 7 and 11 months PI, xenodiagnosis with *I. scapularis* ticks was performed. Serum was collected throughout the study period and tested for reactivity against the C6 peptide. Following euthanasia, tissues were cultured and tested by PCR and RT-PCR for detection of spirochetes and spirochetal nucleic acids.

### Animals, spirochetal inoculation, animal groups, and antibiotic treatment

#### Experiment 1

Twenty-eight rhesus macaques (*Macaca mulatta*) of Chinese origin were used in this study. Their median age when assigned to the study was 2.22 years (range 1.25–4.32). Three of the animals were females and the rest males. All were singly caged, fed commercial monkey chow (Purina Mills 5037 R), and had water available *ad libitum*.

For inoculation, *B. burgdorferi* spirochetes of the JD1 strain [49] were grown in BSK-H medium (Sigma-Aldrich, St. Louis, MO) up to stationary phase (∼10^8^ cells per mL). Twenty-four animals were given an inoculation of 3.2×10^8^ spirochetes each via needle and syringe, with 10^8^ organisms given intraperitoneally in the lower right quadrant, 10^8^ subcutaneously in the same injection site, and 2×10^7^ intradermally, also in the same site. An additional 10^8^ spirochetes were given subcutaneously in the dorsal cervical midline. Four animals were sham-inoculated as uninfected controls. The inoculation and treatment design is summarized in Figure 1A. At 27 weeks PI, 12 of the infected animals and two of the controls were treated with ceftriaxone, followed by doxycycline. Animals received intravenous ceftriaxone, 25 mg/kg, once per day for 30 days (Ceftiofur sodium, Pfizer Animal Health, Kalamazoo, MI) followed by 60 days of oral doxycycline, 2 mg/kg, twice per day (Bio-Serv, Frenchtown, NJ). Twelve infected and 2 control animals were sham-treated. Animal euthanasia began approximately six months post-termination of treatment. Within an additional six-month period all of the animals had been euthanized. The complete time line of the study is depicted in Figure 1B.

#### Experiment 2

Five male rhesus macaques (Chinese origin), 3–4 years of age, were used for the second experiment (outlined in Figure 4C). Four animals were pair-housed after observation under protected contact, and one (GB56) was housed alone. Each animal was given 3 inoculations in the ventral midline of *in vitro*-cultured late log-phase *B. burgdorferi* strain B31, isolate 5A19 [50]: two subcutaneous 1.0 mL injections and one intradermal 0.1 mL injection, each containing 1×10^8^ organisms diluted in sterile Hanks Balanced Salt Solution (HBSS), for a total inoculum of 3×10^8^.

At four months PI, three of the five animals received antibiotic treatment. Animals were trained to accept treats in which the tablets were concealed. Each of the three treated macaques was given one 50 mg tablet of doxycycline (Bio-Serv) twice/day for 28 consecutive days. This dose corresponded to >12 mg/kg/day to ensure that an effective blood level was achieved.

### Determination of the serum concentration of doxycyline *in vivo*

In vitro-cultured *B. burgdorferi* do not grow well in the presence of monkey serum, so antibiotic concentrations in serum had to be tested with alternative reporter strains of bacteria. A 0.5 McFarland suspension of bacteria (*Staphylococcus aureus*, ATCC #29213 or *Bacillus subtilis*, ATCC #6633), made in Mueller-Hinton broth (BD, Franklin Lakes, NJ), was streaked onto Mueller-Hinton agar plates (BD). The streaking was done so that a confluent “lawn” of bacteria would grow after 18–24 hours of incubation at 37°C. To determine antibiotic concentration in serum, 50 µL of serum from treated animals was applied to 6-mm diameter plain paper discs (BD) and allowed to dry for 30 minutes. Standards were made by dissolving doxycycline hyclate (Sigma-Aldrich) into normal monkey serum; these were applied to the discs in the same manner. Standard doxycycline concentrations were 2, 5 and 10-fold the *B. burgdorferi* minimum inhibitory concentration (MIC) when *S. aureus* was used, and 0.5, 1, and 2-fold the MIC when *B. subtilis* was used. Both indicator strains were used in order to expand the range of detection. The doxycycline MIC for the JD1 strain of *B. burgdorferi* was determined to be 0.310 mg/L, which was approximately the same as the mean value of 0.327 mg/L that had previously been obtained for several strains and species of *B. burgdorferi* sensu lato [27].

The test discs were placed onto the prepared agar plates and incubated overnight at 37°C. The zones of inhibition were measured in radial mm. A standard curve was generated using the measurements of the control/standards. The test measurements were compared to the standard curve giving an approximate doxycycline concentration from the test serum. Serum from treated animals was obtained both at the times of peak and trough concentrations for doxycycline, namely, at 2 h and 12 h post-dose administration, during the 8^th^ week of doxycycline treatment. Peak and trough times for rhesus macaques were as per Kelly et al. [51], who in turn based their estimations on pharmacokinetic data from humans.

### Culture of skin biopsy and organ specimens

In both experiments, 4 mm skin biopsy specimens were collected from all animals, under anesthesia, weekly during the first four weeks PI and were cultured in BSK-H medium as described [11]. Skin biopsy samples were thus collected from live animals before antibiotic treatment so as to confirm infection. Organ specimens were also collected at necropsy. In Experiment 1, the following organ specimens, obtained postmortem, were cultured: brain (20 samples per monkey), meninges (10), dorsal root ganglia (5), sciatic nerve (10), skin (10), heart (10), synovial membrane (2), peritoneal membrane (10), lung (5), bladder (5), and spleen (5). In Experiment 2, specimens of skin, heart, bladder, spleen, knee joint synovial membrane, and shoulder tendon were cultured. Two ∼2 mm^3^ tissue sections from each organ were cultured.

### Detection of spirochetal DNA

In Experiment 1, real time PCR targeted at the *ospA* and *flaB* genes was performed following a protocol described previously [52]. DNA extracted from up to ten specimens each of brain, meninges, dorsal root ganglia, sciatic nerve, skin, heart, synovial membrane, peritoneal membrane, lung, bladder, and spleen was amplified for all animals postmortem. Only those specimens that were positive for both the *ospA* and *flaB* genes were considered positive.

In Experiment 2, standard PCR was used for detection of *B. burgdorferi* from tick midguts, culture pellets and animal tissues. DNA was extracted from ∼60 mg (30 mg×2) of each tissue (heart, bladder, spleen, knee joint synovium, shoulder tendon and skin) with the DNeasy® Blood and Tissue kit (Qiagen, Valencia, CA). Primer sets that target the *flaB* and *ospC* genes were used and are published [53]. The Taq DNA polymerase Core Kit (Qiagen) was utilized according to the manufacturer's protocol. For each primer, 0.4 µM concentration was used. PCR was performed with 35 cycles of denaturation (94°, 30 sec.), annealing (43–53°, 45 sec.) and extension (72°, 1 min.). Negative controls included DNA extracted from uninfected monkey tissue (from Experiment 1) and DNA extracted from a pool of 5 ticks derived from the same egg mass as those used for xenodiagnosis.

### Detection of spirochetal transcripts by reverse transcriptase PCR

For Experiment 1, total RNA from heart and brain specimens from all of the animals at postmortem was isolated using the Trizol method (Invitrogen, Carlsbad, CA) and contaminating DNA was removed from 2–5 µg of total RNA specimens using the DNA-free™ kit, according to the manufacturer's protocol (Applied Biosystems/Ambion, Austin, TX). cDNA was subsequently synthesized from 1 µg of DNA-free RNA using the iScript cdNA synthesis kit (Bio-Rad, Hercules, CA). No amplicon was obtained by the procedure described below when the cDNA synthesis step was omitted. One µl of cDNA from each of the monkey tissue samples served as template to PCR amplify a 275 bp fragment of the *B. burgdorferi* flagellin (*flaB*) transcript. The forward primer was 5′-TTGCTGATCAAGCTCAATATAACCA-3′ and the reverse primer was 5′-CACCGGTTCAAGAGGGTGTT-3′. PCR was performed using Taq polymerase (Roche, CA). The cycling conditions were as follows: 1 cycle of denaturation at 94°C for 3 min followed by 35 cycles of 30 sec denaturation at 94°C, 30 sec annealing at 60°C and 30 sec extension at 72°C and one cycle of 5 min extension at 72°C. PCR products were electrophoresed on a 1.5% agarose gel and the gel processed for Southern blotting as described [54]. Separated amplicons were capillary transferred to Hybond N+ nitrocellulose membrane (Amersham Biosciences, Buckinghamshire, UK) as described [54]. A 22-mer oligonucleotide corresponding to a sequence within this amplicon (5′-CTGCTTCTCAAAATGTAAGAACAGCTGAAGAGCTTG-3′) was synthesized at the W. M. Keck Facility at Yale University and non-radioactively labeled using the Gene Images AlkPhos Direct Labeling and Detection system (Amersham Biosciences, UK). The labeled probe was hybridized to the nitrocellulose membrane at 55°C overnight and washed as described in the manual (Amersham Biosciences, UK). Hybridization and washings were performed in hybridization bottles in a hybridization oven (Labnet International, Woodbridge, NJ). Bound probe was detected using the CDP-*Star* detection reagent for chemiluminescent detection of alkaline phosphatase (Amersham Biosciences, UK). Two independent RNA preparations were obtained from heart and brain tissues. Tissue specimens were considered positive only when they were positive in both preparations.

In Experiment 2, standard RT-PCR detection was used. RNA was extracted from the tissues with the RNeasy® Fibrous Tissue Mini Kit and treated with the RNase-free DNase set (Qiagen) to remove any residual DNA. The *ospA* and *bbf26* genes were amplified, using the following primers: (1) *ospA* forward primer 5′-AATGTTAGCAGCCTTGACGA-3′; reverse primer 5′-TCGTACTTGCCGTCTTTGTT -3′; (2) *bbf26* forward primer 5′-TGCCTCTAATTGTGAACACC-3′; reverse primer 5′-TCAAATCTTGAACAATACACTCA-3′. RT-PCR was performed with the Qiagen® OneStep RT-PCR kit. A quantity of 100–250 ng total RNA was used as template, with primer concentrations of 0.8 µM (forward) and 1.2 µM (reverse). The cycling was carried out per manufacturer's suggestion with the GeneAmp PCRSystem 9700 (Applied Biosystems, Carlsbad, CA). Each set produces a ∼100 bp amplicon. Samples of RNA from monkey tissue and *B. burgdorferi* cultures were tested for DNA contamination by PCR amplification using *Taq* polymerase and none was detected (Figure S2).

### Detection of anti-C6 antibody

Anti-C6 serum antibody levels were quantified in all animals starting prior to inoculation and then serially thereafter until post-euthanasia. The ELISA procedure used has been described [55]. In Experiment 1, monkey serum was used at a dilution of 1∶800 for all time points. The cut-off ELISA value for each plate was set as the mean of the optical density at 450 nm (OD_450_) of duplicate determinations of pre-immune serum specimens from 7 animals plus 3× the standard deviation (SD). To correct for plate-to plate variations, a reference positive control serum pool was also included in each plate, in triplicate. This pool was made by combining equal volumes of serum that was obtained from 24 animals inoculated with *B. burgdorferi*. The aliquots for the pool were from serum specimens that were obtained from each animal on two consecutive collection dates just prior to the initiation of antibiotic treatment. The mean OD_450_ of the triplicate determination for each plate was used as a divisor for all of the OD_450_ values obtained with that plate, including the cut-off value, and values were reported as a quotient (index).

In Experiment 2, the same peptide antigen (C6), here derived from the strain B31 sequence, and ELISA procedure as in Experiment 1 was used. However, all responses could be compared on one plate, so the conversion to an index value was not required. Blood was collected at the following time points: 0, 2, 6, 10, 14, 16, 18, 22, 26, 28, 34, 40 and 47 weeks PI. Monkey serum was used at a 1∶400 dilution for standard ELISA and at 2-fold dilution (in 5% pre-immune serum) from 1∶40 through 1∶2560 for endpoint dilution titers. The mean OD_450_ value of triplicate pre-immune serum samples from each individual animal was subtracted from the value at each time point or dilution for that animal. The reported end-point dilution titer is the dilution at which the sample reached the highest OD_450_ value for pre-immune serum+2× the SD obtained from that animal. In addition, serum from 2 weeks PI was tested for antibody to the Ct peptide [56] and OspC by ELISA, as described [56], [57]. Recombinant OspC was kindly provided by Robert Gilmore (CDC, Fort Collins, CO).

### Detection of antibody to *B. burgdorferi* whole-cell antigen extract (Experiment 1)

*B. burgdorferi* antigen was extracted from spirochete cells by sonication in PBS, and dispensed onto 96-well ELISA plates (Corning® Costar®, Lowell, MA) at 100 µL/well (0.1 µg protein) in 50 mM carbonate buffer pH 9.6 overnight at 4°C. Plates were washed with 0.1% Tween 20 in PBS (PBS/T) and blocked for 1 h with 200 µL of 5% nonfat dried milk in PBS/T in a rotating plate set at 100–150 rpm. Serum specimens from bleeds obtained prior to inoculation, immediately prior to antibiotic treatment initiation, and at necropsy were diluted in PBS/T and tested at dilutions of 1∶800 and 1∶3200. Antibody bound to the plate was reacted with horseradish peroxidase-labeled goat anti-monkey IgG (gamma chain-specific, Kirkegaard and Perry Labs, (KPL), Gaithersburg, MD) at a dilution of 1∶5000, for 1 h, followed by TMB Microwell Peroxidase Substrate System (KPL). The color reaction was allowed to proceed for 8–9 min, and stopped by adding 100 µL per well of 1.0 M phosphoric acid. OD_450_ was measured on a plate reader (Biotek Instruments, Winooski, VT).

### Gross pathology assessment (Experiment 1)

Several organs were assessed for gross pathology by a veterinary pathologist, including heart, lungs, spleen, liver, musculoskeletal system, bladder, kidneys, peripheral lymph nodes, brain and meninges.

### Detection of inflammatory lesions and spirochetal antigen in tissues (Experiment 1)

To detect inflammatory lesions in heart and meninges that were collected postmortem, tissue fragments were fixed in Z-fix (Anatech Ltd., Battle Creek, MI), embedded in paraffin, sectioned at 6 µm, and stained with hematoxylin and eosin. Stained sections were evaluated under a light microscope using 4–40× magnification and a Leica microscope with a SPOT Insight digital camera (Digital Instruments Inc., Michigan, USA). To detect spirochetal antigen a monoclonal antibody (MAb 240.1) was used. This IgG1 MAb reacts with a 7.5 kDa *B. burgdorferi* lipoprotein [58] and had been employed before by us to detect *B. burgdorferi* antigen by immunohistochemistry [13]. Deparaffinized sections were washed with PBS containing 0.2% fish-skin gelatin and 0.1% Triton X-100 (PBS-FSG-Tx100) at room temperature for 10 minutes. Sections were then blocked with 10% normal goat serum (NGS) diluted in phosphate buffered saline containing 0.2% fish skin gelatin (PBS-FSG) in a humidified chamber, also at room temperature, for 40 minutes. The primary antibody was diluted in NGS and incubated for 1 hour at room temperature in a humidified chamber protected from light. Following incubation the slides were washed twice with PBS-FSG-Tx100 for ten minutes each. *B. burgdorferi* immunofluorescence was revealed using a species-specific goat anti-mouse secondary antibody coupled with Alexa 488 (Molecular Probes, Eugene, OR). The secondary antibody was also diluted in 10% NGS and incubated in a humidified chamber for 40 minutes at room temperature protected from light. Following incubation, the slides were washed twice with PBS-FSG-Tx100 for ten minutes each and followed with PBS-FSG. Upon completion of immunofluorescence staining, the sections were rinsed in doubly distilled water and mounted in an anti-quenching solution composed of 0.33 g/mL glycerol, analar grade, 0.133 g/mL MOWIOL 4–88 (Calbiochem), 33.33% double distilled water, 66.66% 0.2 M Tris Buffer, and 25 mg/mL DABCO (1.4-diazobicyclo-[2.2.2.]-octane) (Sigma-Aldrich) and coverslipped.

### Xenodiagnosis

In Experiment 2, xenodiagnosis was performed at 7 and 11 months PI, which translated to 2 and 6 months after cessation of treatment. At 27 weeks PI, nylon-mesh jackets that zip in the back (Lomir Biomedical, NY) were placed on each of the five animals for acclimatization. At 28 weeks PI, 10 laboratory-reared nymphal *Ixodes scapularis* ticks were placed on the back of each animal through a containment capsule by a procedure that has been described [11]. Briefly, the area of the back to be used for tick placement was shaved and wiped with SkinPrep (Smith & Nephew Medical, UK). The tick containment capsule was fixed to the skin at its base with Super Glue and at the edges with SkinBond contact adhesive (Smith & Nephew Medical). The capsule was further secured into place with Hypafix tape (Smith & Nephew Medical). The ticks were placed inside the capsule on the skin and sealed over with a piece of nylon mesh and screw cap lid, so that ticks could not escape from the capsule. The average feeding time for ticks is 72 hours, so to allow time for them to feed to repletion, they were removed 4 days later. For tick removal, the capsules were opened and ticks were collected with a paintbrush. Ticks that had not detached from the skin were removed carefully with forceps. The capsules were gently removed by dissolving the adhesive with UniSolve (Smith & Nephew Medical) so as not to irritate the skin.

Ticks were washed with 1% sodium hypochlorite, 0.5% benzalkonium chloride and 70% ethanol for 1 min each and crushed in 45 µL PBS with a microfuge pestle. The contents were split into three 15-µL portions for: (1) direct fluorescence assay (DFA); (2) culture; and (3) PCR. For DFA, the samples were smeared on microscope slides, dried and fixed with acetone. The samples were stained with an anti-*Borrelia*-FITC antibody (KPL) and examined by fluorescence microscopy to detect the presence of spirochetes [59]. For culture, samples were added to ∼4 mL BSK-H medium and incubated for 8 weeks at 34°C, in the presence of 5% CO_2_ and influx of N_2_ to produce a microaerophilic environment.

### Statistical analysis (Experiment 1)

Differences between treated and untreated animals in detection results of spirochetal DNA, RNA, antigen, and in the C6 antibody response at postmortem were evaluated with Fisher's exact test (two-tailed). Differences were considered significant with p≤0.5.

## Supporting Information

Table S1 **Evidence of Productive Infection in Experimental Animals (Experiment 2).**
(DOC)

Table S2 **Xenodiagnostic Ticks Recovered (Experiment 2).**
(DOC)

Figure S1 **The decline in C6 antibodies that accompanied antibiotic treatment in Experiment 2.** Animal designations in **bold** (black lines) indicate those that were treated. Antibody levels indicated by gray lines are from the untreated animals.
(TIF)

Figure S2 **RNA samples tested for DNA contamination with** ***Taq*** **polymerase PCR.** Samples included the 100 bp ladder (M), no template control (ntc), *B. burgdorferi* DNA (Bb DNA), *B. burgdorferi* RNA (Bb RNA), monkey DNA (Mk DNA) and monkey RNA (Mk RNA).
(TIF)
