# Correction: Persistence of *Borrelia burgdorferi* in Rhesus Macaques following Antibiotic Treatment of Disseminated Infection

**DOI:** 10.1371/annotation/4cafed66-fb84-4589-a001-131d9c50aea6

**Published:** 2012-04-09

**Authors:** Monica E. Embers, Stephen W. Barthold, Juan T. Borda, Lisa Bowers, Lara Doyle, Emir Hodzic, Mary B. Jacobs, Nicole R. Hasenkampf, Dale S. Martin, Sukanya Narasimhan, Kathrine M. Phillippi-Falkenstein, Jeanette E. Purcell, Marion S. Ratterree, Mario T. Philipp

There was an error in the Materials and Methods section titled Animals, spirochetal inoculation, animal groups, and antibiotic treatment. The correct text is "(Rocephin, ceftriaxone sodium, Hoffmann - La Roche Inc.)". 

