# Correction: Persistence of *Borrelia burgdorferi* in Rhesus Macaques following Antibiotic Treatment of Disseminated Infection

**DOI:** 10.1371/annotation/f84663e3-0a2c-4243-8f97-3a58133c1b0f

**Published:** 2013-09-24

**Authors:** Monica E. Embers, Stephen W. Barthold, Juan T. Borda, Lisa Bowers, Lara Doyle, Emir Hodzic, Mary B. Jacobs, Nicole R. Hasenkampf, Dale S. Martin, Sukanya Narasimhan, Kathrine M. Phillippi-Falkenstein, Jeanette E. Purcell, Marion S. Ratterree, Mario T. Philipp

In the Results section of the article, the first paragraph under the heading "Monitoring of infection status postmortem." had an error in its last sentence as a result of issues in the typesetting process. The correct sentence is available below.

"With regard to culture of spirochetes, none of the treated animals yielded a positive culture and only one of the untreated animals was culture-positive (lung tissue)." 

