# Supplementary material for: Persistence of Borrelia burgdorferi in Rhesus Macaques following Antibiotic Treatment of Disseminated Infection
Source: PLoS One. 2012 Jan 11;7(1):e29914. doi: 10.1371/journal.pone.0029914 (PMC3256191; doi:10.1371/journal.pone.0029914)
Supplement: Table S1 — Evidence of Productive Infection in Experimental Animals (Experiment 2). (DOC) [file pone.0029914.s001.doc]

**Table S1**

|  | **Skin biopsy PCR (OspC)** | **Culture** | **C6 ELISA**  **(6wk.)** | **Ct ELISA**  **(6 wk.)** | **OspC ELISA**  **(2 wk.)** |
| --- | --- | --- | --- | --- | --- |
| **GA59** | + + + - | - - - - | + | + | + |
| **FK38** | + - - - | - - - - | + | + | + |
| **GC84** | + - - - | - - - - | + | + | - |
| **FT47** | + - + - | - - - - | + | + | + |
| **GB56** | + + + + | - - - - | + | + | + |
