# Supplementary material for: Persistence of Borrelia burgdorferi in Rhesus Macaques following Antibiotic Treatment of Disseminated Infection
Source: PLoS One. 2012 Jan 11;7(1):e29914. doi: 10.1371/journal.pone.0029914 (PMC3256191; doi:10.1371/journal.pone.0029914)
Supplement: Table S2 — Xenodiagnostic Ticks Recovered (Experiment 2). (DOC) [file pone.0029914.s002.doc]

**Table S2**

|  | **Xeno #1 (7 mo.)** | **Xeno #2 (11 mo.)** | **Total** |
| --- | --- | --- | --- |
| **GB56** | 11 | 5 | 16 |
| **GA59** | 8 | 1 | 9 |
| **FK38** | 7 | 2 | 9 |
| GC84 | 5 | 2 (partially-fed) | 7 |
| FT47 | 5 | 1 | 6 |
