# Supplementary figures and images for: Persistence of Borrelia burgdorferi in Rhesus Macaques following Antibiotic Treatment of Disseminated Infection
Source: PLoS One. 2012 Jan 11;7(1):e29914. doi: 10.1371/journal.pone.0029914 (PMC3256191; doi:10.1371/journal.pone.0029914)

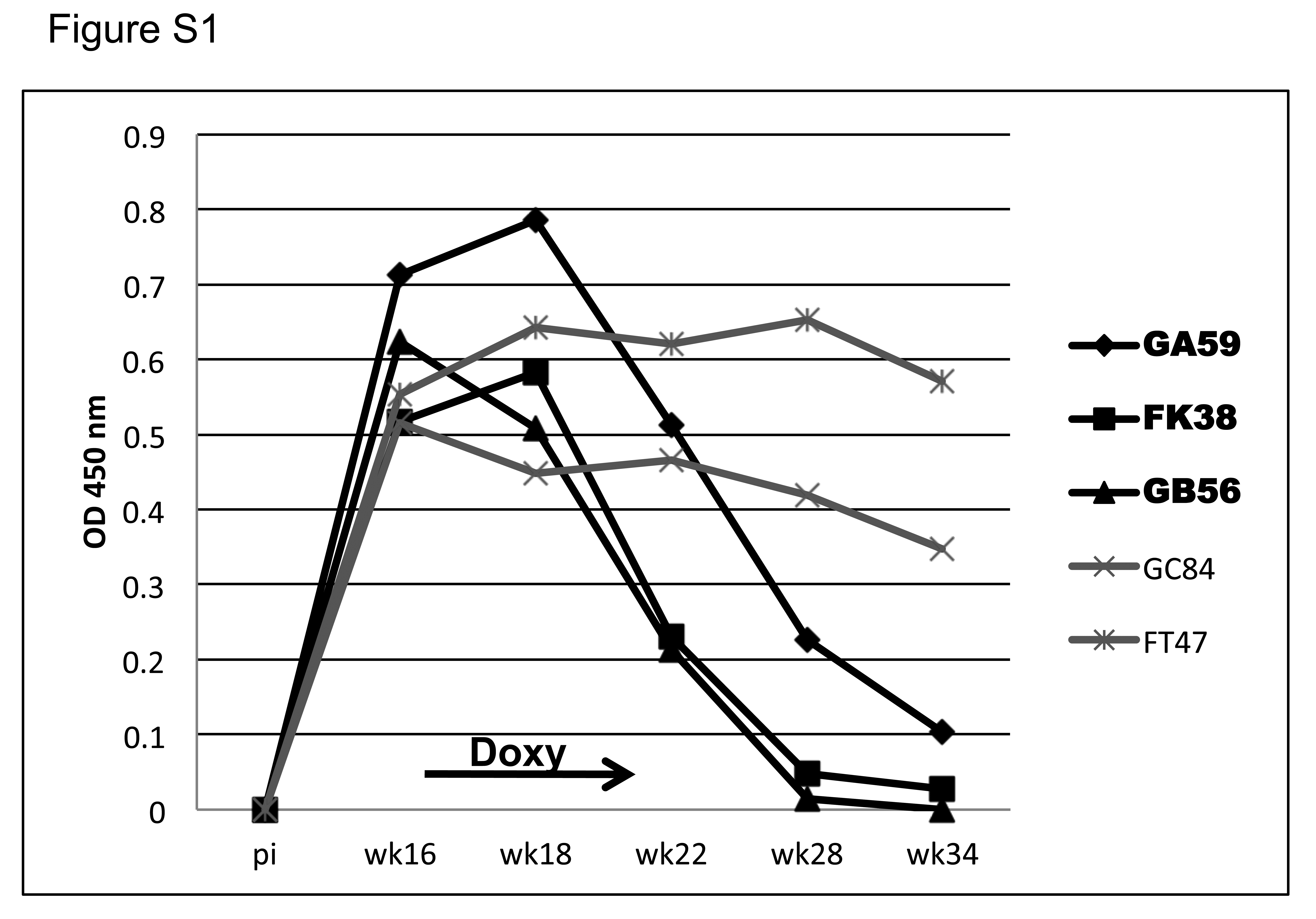

Supplement: Figure S1 — The decline in C6 antibodies that accompanied antibiotic treatment in Experiment 2. Animal designations in bold (black lines) indicate those that were treated. Antibody levels indicated by gray lines are from the untreated animals. (TIF) [file pone.0029914.s003.tif]

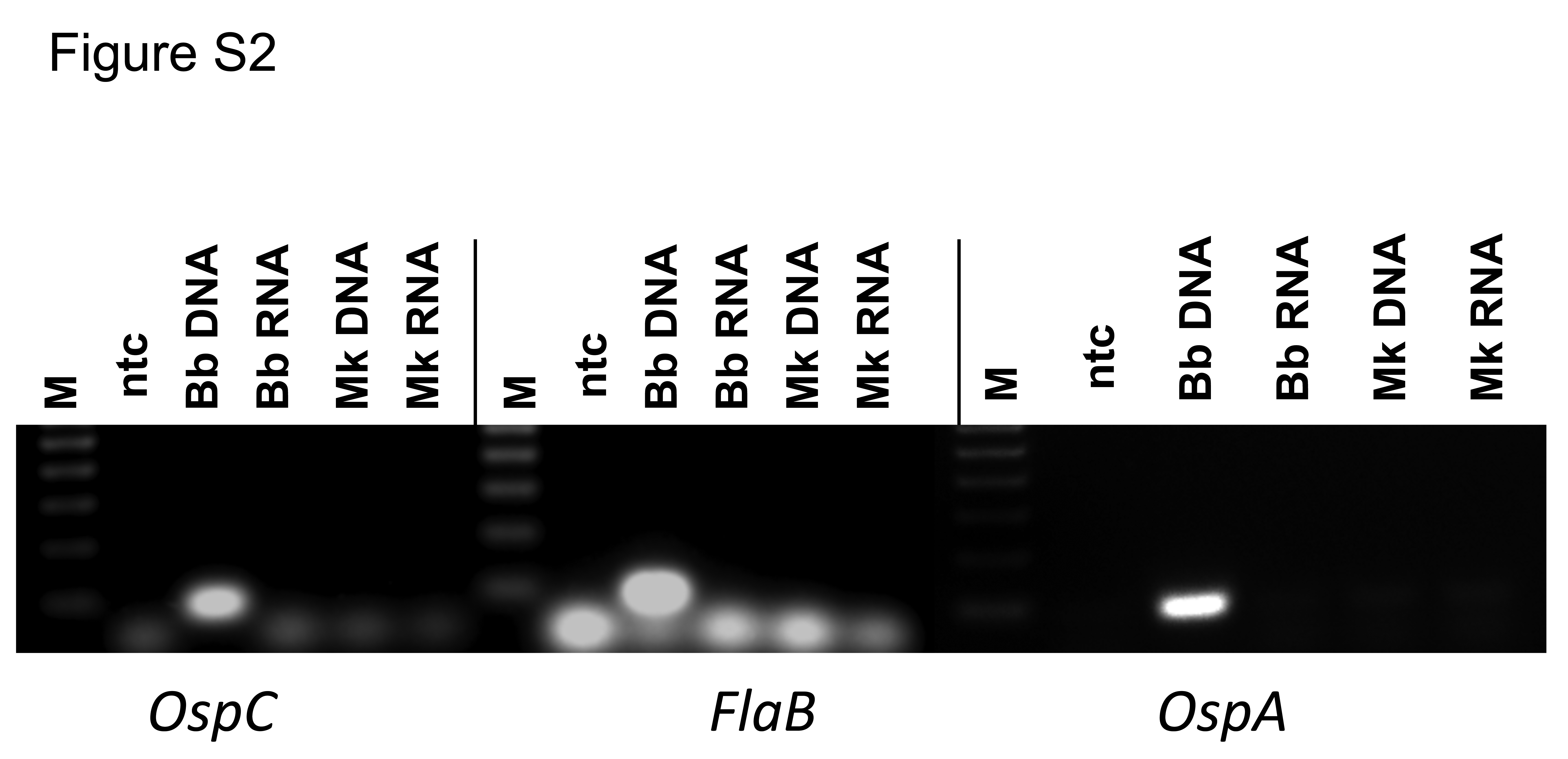

Supplement: Figure S2 — RNA samples tested for DNA contamination with Taq polymerase PCR. Samples included the 100 bp ladder (M), no template control (ntc), B. burgdorferi DNA (Bb DNA), B. burgdorferi RNA (Bb RNA), monkey DNA (Mk DNA) and monkey RNA (Mk RNA). (TIF) [file pone.0029914.s004.tif]
